# A comparative multi-parametric *in vitro* model identifies the power of test conditions to predict the fibrotic tendency of a biomaterial

**DOI:** 10.1038/s41598-017-01584-9

**Published:** 2017-05-10

**Authors:** Maren Jannasch, Sabine Gaetzner, Tobias Weigel, Heike Walles, Tobias Schmitz, Jan Hansmann

**Affiliations:** 1 University Hospital Wuerzburg, Department Tissue Engineering and Regenerative Medicine (TERM), Roentgenring 11, 97070 Wuerzburg, Germany; 2 Fraunhofer Institute for Interfacial Engineering and Biotechnology, Translational Center Wuerzburg Regenerative Therapies in Oncology and Musculoskeletal Disease, 97070 Wuerzburg, Germany

## Abstract

Despite growing effort to advance materials towards a low fibrotic progression, all implants elicit adverse tissue responses. Pre-clinical biomaterial assessment relies on animals testing, which can be complemented by *in vitro* tests to address the Russell and Burch’s 3R aspect of reducing animal burden. However, a poor correlation between *in vitro* and *in vivo* biomaterial assessments confirms a need for suitable *in vitro* biomaterial tests. The aim of the study was to identify a test setting, which is predictive and might be time- and cost-efficient. We demonstrated how sensitive *in vitro* biomaterial assessment based on human primary macrophages depends on test conditions. Moreover, possible clinical scenarios such as lipopolysaccharide contamination, contact to autologous blood plasma, and presence of IL-4 in an immune niche influence the outcome of a biomaterial ranking. Nevertheless, by using glass, titanium, polytetrafluorethylene, silicone, and polyethylene representing a specific material-induced fibrotic response and by comparison to literature data, we were able to identify a test condition that provides a high correlation to state-of-the-art *in vivo* studies. Most important, biomaterial ranking obtained under native plasma test conditions showed a high predictive accuracy compared to *in vivo* assessments, strengthening a biomimetic three-dimensional *in vitro* test platform.

## Introduction

The increasing use of biomaterials in regenerative medicine to replace or repair tissue defects or to support body functions, demonstrates the clinical relevance of appropriate biocompatibility test methods. Following implantation, all biomaterials induce adverse tissue responses, comprising inflammation, fibrosis or thrombosis^1–3^. Upon exposition of a biomaterials’ surface to blood fluids, proteins adsorb immediately, thereby enabling cell adhesion, coagulation, and complement activation. Induced by tissue injury and affected by protein-material interactions, alarm signals are released to the extracellular space, stimulating the migration of immune cells to the implant region^4, 5^. Within hours, granulocytes enter the tissue site and start to structurally attack by the release proteases and reactive oxygen species the surrounding tissue and the biomaterial. Following next days, granulocytes are replaced by macrophages at the wound site^6^. Usually, implant dimension and a non-degradable nature cause a failure to clearance the foreign body by phagocytosis^7^. The persistence of macrophages in direct contact to the implant surface confirms the central role of macrophages in the foreign body reaction. Over time, a transition from short-lived pro-inflammatory M1 to long-vitae M2 macrophages is observed^8, 9^. This cellular polarization is influenced by inflammatory mediators, such as material-adhesive, pathogen-associated molecular patterns or local cytokine milieu^10^. For example, an inadequate cleaning of the implant can be apparent by a surface contamination with lipopolysaccharide (LPS), an outer membrane component of Gram-negative bacteria, shifting cellular balance to a pro-inflammatory state^11^. In contrast to that, IL-4, produced by granulocytes or T_H_2-lymphocytes, strengthens the longevity of M2 macrophages on biomaterial surface by induction of multi-cellular membrane fusion to giant cells^12, 13^. Furthermore, systemic conditions such as alterations in metabolism, e.g. diabetes and smoking habits, as well as quantity and quality of tissue contribute to individual compatibility reactions between recipients^14–16^. The cellular shift towards M2 phenotype is one key step towards cellular isolation of the foreign body from internal host’s body. The chronic deposition of M2 macrophages in the proximity of the implant stimulates surrounding fibroblasts to generate a dense fibrous capsule^8, 9^ – exemplarily demonstrated by M2-phenotypic arginase expression metabolizing arginine to ornithine, which in turn promotes fibrosis by proliferation and collagen synthesis^17, 18^. To overcome those fibrotic adverse side effects and thereby ensure therapeutic success constitute main challenges in biomaterial development.

Current gold standard for the assessment of material-induced local tissue responses after implantation are animal studies [ISO 10993-6:2007, Part 6]. In complement to those long-term *in vivo* studies, *in vitro* tests facilitate short-term screening for acute effects of blood-material interaction [ISO 10993-4:2002, Part 4]. However, as inadequate test conditions often result in a poor correlation between *in vitro* and *in vivo* ^19^, it must be ensured that obtained *in vitro* results are not biased or even determined by the test conditions or performance of experimental setup. To achieve an *in vitro* test that correlates to the fibrotic response observed *in vivo*, our aim was to identify test conditions for the development of a predictive human *in vitro* test system. Such an *in vitro* test system might be more efficient in terms of cost and time than animal testing and aligns to the 3R’s principle to reduce animal burden^20^.

**Figure 1:**
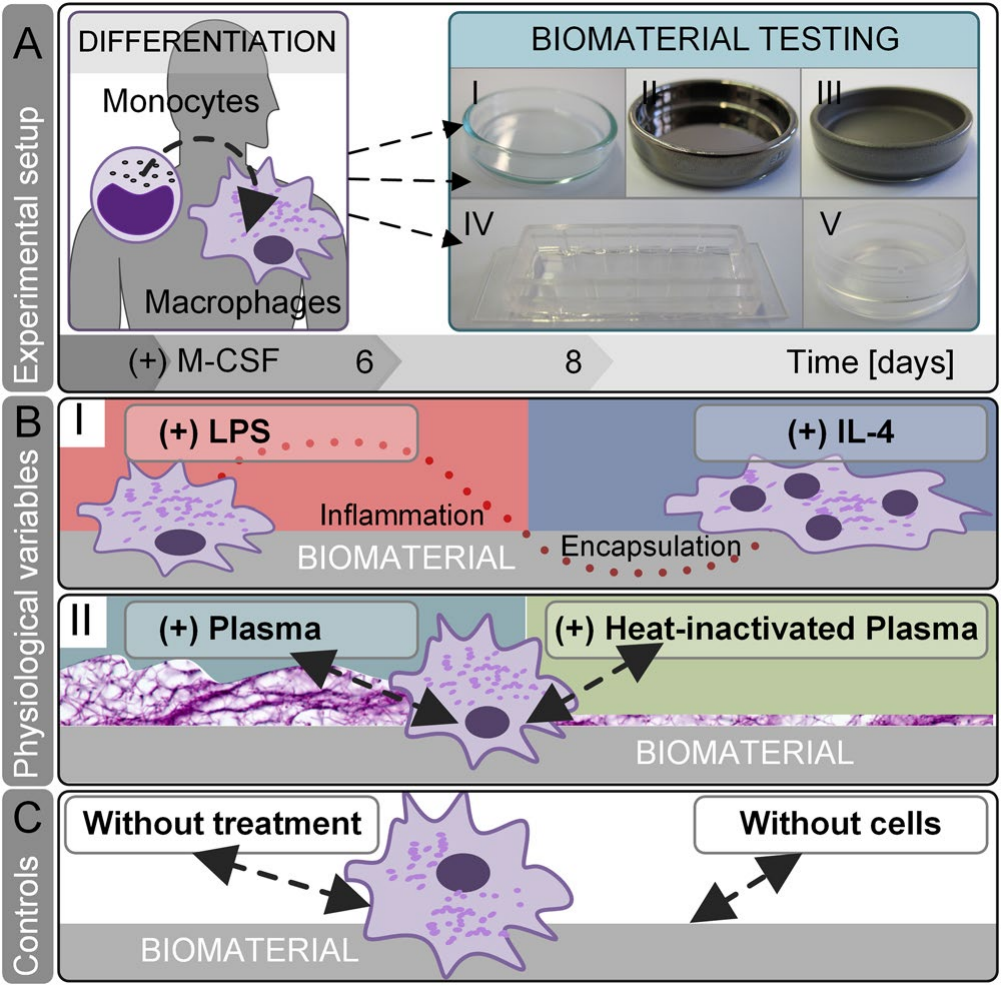
*In vitro* screening of physiological test conditions for the predictive power to evaluate a biomaterials’ fibrotic potential. (**A**) In our experimental setup, human blood-derived monocytes were differentiated by supplementation of macrophage colony-stimulating factor (M-CSF) to M2-like macrophages. Subsequently, macrophages were cultured on biomaterials for 48 h: (I) glass, (II) titanium, (III) PTFE, (IV) silicone and (V) PE. (**B**) On each material, we tested conditions that mimic the physiological *in vivo* niche on materials’ surface: (I) A common cause of implant failure - LPS contamination - polarizes macrophages’ fate towards pro-inflammation. In contrast, the presence of IL-4 in the immune niche strengthens a pro-survival cellular phenotype – the fusion of macrophages towards foreign body giant cells. (II) A biomimetic approach of protein-material interaction was resembled by applying human autologous blood-derived plasma on biomaterials surface. By calcification of plasma, a primary fibrous three-dimensional niche was formed. In comparison to native blood plasma, the inactivation of heat labile protein, e.g. complement, growth, and coagulation factors was assessed by heat-inactivation (HI) of human plasma. (**C**) As controls served test conditions without any additions and without cells.

Beside technical test variables like surface-to-volume ratio, sample collection, or tested readout parameters, also physiological variables recapitulating the process of foreign body reaction influence the materials’ assessment *in vitro*, e.g. the interaction of blood plasma proteins with a biomaterial surface. Our experimental approach (Fig. 1) assesses the impact of physiological test conditions for the development of a standardized and predictive *in vitro* biomaterial test system. Those test conditions reflect a biomimetic implant scenario such as implant contamination, an immunological wound niche and blood protein-material interaction.

To consider that monocytes differentiate *in vivo* immediately upon extravasation, our testing was based on monocyte-derived primary human macrophages. Although this mechanism is important, the differentiation of monocytes into a M2-like phenotype prior to biomaterial testing is often neglected. These cells were used in a specific inflammatory spectrum, which moreover mimic the heterogeneous human immune system closer than immortalized cell lines. Due to its involvement in the long-term fibrotic response to biomaterials, macrophages were differentiated towards a M2-like phenotype by applying macrophage colony-stimulating factor (M-CSF) to the culture medium^21, 22^. LPS and IL-4 test conditions allowed modeling a broad inflammatory spectrum of macrophages: LPS induces a strong inflammation that polarizes a M2-like-to-M1 transition, whereas IL-4 strengthens a M2-phenotypic escape of apoptosis towards cellular adherence and fusion^23^.

To ensure that the test system is applicable for a broad spectrum of biomaterials, we tested five distinct types of materials: glass, titanium, polytetrafluorethylene (PTFE), silicone, and polyethylene (PE). In regenerative medicine, titanium is preferably used in bone replacement^24–26^, whereas PTFE is applied for vascular prosthesis, nerve conduits, or subcutaneous augmentation^27, 28^. Silicone is commonly harnessed for soft tissue replacement^29^, e.g. as mammary implant or in laryngoplasty^30^, and PE is found in joint replacement^31, 32^, facial skeletal or head augmentation^33, 34^. We additionally included glass as a control for a standard cell culture material. In order to allow an evaluation of the fibrotic tendencies of those materials, we reviewed literature on animal and clinical studies (see Table 1). Although no meta-analysis of the tested biomaterials is available, several preclinical and clinical studies allow a comparison and ranking of the used materials respective to their fibrotic potential. A good long-term survival, tissue ingrowth and a low complication incidence is shown for PE implants in humans^32–34^. In animal studies, also titanium was characterized by a low fibrotic response^35–38^, whereas a moderate inflammatory reaction and a fibrous encapsulation were observed following PTFE implantation^38, 39^. Strongest adverse effects such as a thick capsule formation were elicited by silicone implants^40–43^. Despite silicone’s endorsement as the most widely applied biomaterial, a debate on its safety in humans continuously remains^29, 44^. These studies demonstrate an increasing fibrotic potential from PE, titanium, PTFE to silicone, and were used for the validation of our test conditions.

**Table 1:** Preclinical and clinical studies allowed a comparison and evaluation of the test materials regarding their fibrotic tendencies.

Year[Reference] Author	Category [species, application]	Type of material	Outcome
2015^32^ Kindsfater	clinical study [human, knee bearing]	Polyethylene	PE showed no revisions, osteolysis or implant dissociation.
2010^33^ Deshpande	clinical study [human, facial skeletal augmentation]	Polyethylene	PE had a good long-term survivorship and a low complication incidence.
1993^34^ Wellisz	clinical study [human, facial or head reconstruction]	porous Polyethylene	Porous PE exhibited tissue ingrowth.
2008^35^ Suska	animal study [rat, subcutaneous implant]	Titanium, cupper	Titanium surrounded a thinner fibrous capsule with lower inflammatory cells and vascularity than cupper.
1994^36^ Ungersböck	animal study [rabbit, tibia implant]	Titanium, stainless steel	Fibrous tissue surrounding titanium was thinner and inflammatory cellular numbers were lower compared to stainless steel.
1997^37^ Shannon	animal study [rats, subcutaneous implant]	Titanium, stainless steel	In between titanium and stainless steel no differences in capsule thickness and cell response were found. Qualitative capsule characterization revealed less dense and circumferentially-packed tissue around titanium compared to stainless steel.
1986^38^ Thomsen	animal study [rat, abdominal wall implant]	Titanium, PTFE	Titanium implants were in direct contact with the connective tissue without inflammatory cells. In contrast, a fibrous capsule surrounded the PTFE implants.
1978^39^ von Recum	animal study [dog, aortic patch]	PTFE, Polyurethan	Polyurethan was encapsulated in a fluid cyst, whereas PTFE was moderately surrounded by tightly adherent fibrous tissue.
2002^40^ Batniji	animal study [rabbit, subperiosteal pocket implant]	PTFE, Silicone	The silicone implants elicited compared to PTFE a significantly thicker capsule and less neovascularization.
2005^41^ Ustundag	animal study [rabbit, paraglottic space implant]	PTFE, Silicone	Around silicone a fibrous capsule formed, whereas PTFE limited the formation of a fibrous capsule.
1996^42^ Trumpy	clinical study [human, subcutaneous implant]	PTFE, hard and soft Silicone	All materials developed a fibrous capsule decreasing in order to soft silicone, PTFE and hard silicone.
2003^43^ Siggelkow	clinical study [human, breast implant]	Silicone	A main reason for explantation of intact implants was capsular contracture, which was related to capsule thickness.

These studies emphasized an increasing fibrotic response from PE, titanium, PTFE to silicone. This literature-based biomaterial ranking finally substantiated the validation of our test conditions on their predictive power.

**Figure 2:**
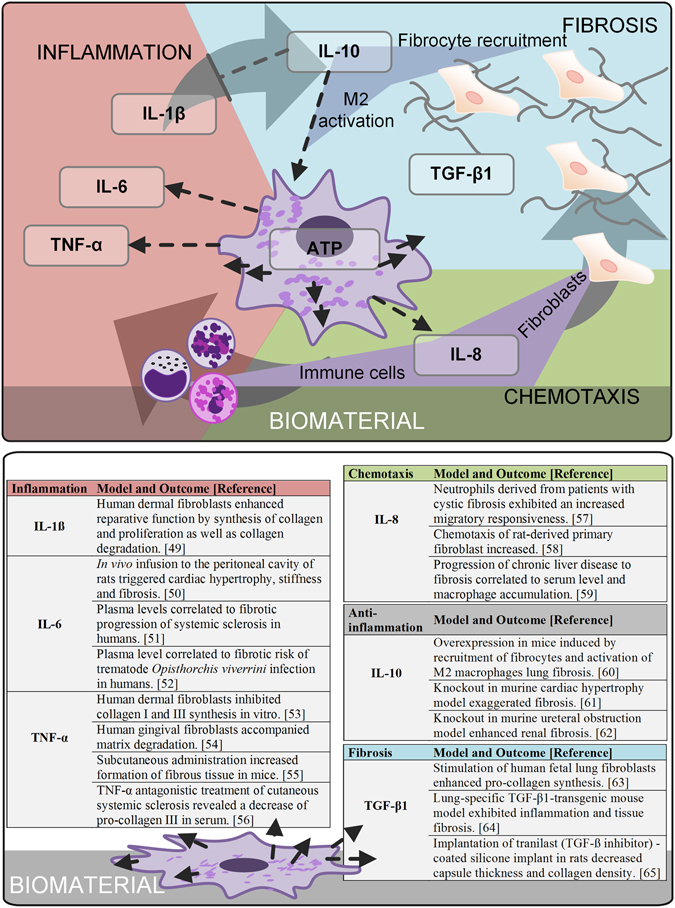
To characterize materials’ fibrotic potential, the influence of the selected test conditions on the net secretion of cytokines was investigated. All selected readout factors represent fibrotic drivers: a strong cellular viability on materials’ surface maintains a chronic responsiveness to the foreign body; a pro-inflammatory component at the initial stage of cell-implant contact induces a fibrotic progression and a chemokine gradient guiding further cells to implant region strengthens both the inflammatory and fibrotic response. Despite the mechanism of surface-induced cytokine secretion and their influence to final fibrotic progression is poorly understood, pre-clinical and clinical studies from adjacent research areas substantiate the selection of readout parameters.

A still newly arising field to asses a material’s biocompatibility is to characterize the potential to induce the secretion of inflammatory mediators^45^. The capacity of materials to modulate the cytokine response has been already demonstrated for murine macrophage-like RAW 264.7 and primary monocytes^46, 47^. However, a mechanistic understanding on biomaterial-surface-induced cytokine secretion and its role in fibrosis remained unresolved until now^45^. Therefore, the influence of the selected test conditions on the cytokine net effect was investigated and caught by multiple readout parameters. Despite the mechanism of surface-induced cytokine secretion and their influence to final fibrotic progression is poorly understood^17^, studies from adjacent research areas substantiate the understanding on cytokines’ involvement in fibrosis (Fig. 2). From state-of-the-art cytokine classifications the following assumptions were made:

-   (I) a strong cellular viability on materials’ surface maintains a chronic responsiveness to the foreign body

-   (II) a pro-inflammatory component at the initial stage of cell-implant contact triggers a fibrotic progression
- (III) a chemokine gradient guiding further cells to implant region strengthens both the inflammatory and the fibrotic response

These assumptions finally rendered the fundament for the selection and interpretation of the tested quantitative readout factors: we quantified the cytokines IL-1ß, IL-6, TNF-α, IL-8, IL-10 and TGF-ß1 in the cell culture supernatant; additionally, ATP levels (CellTiter) considered cellular viability at the surface site.

To test pro-inflammatory cytokines such as IL-1ß, IL-6 and TNF-α at short-term 48 hours addresses the initial inflammatory trigger of a fibrotic progression^48^. Whereas IL-1ß shows unique bi-functional reparative- as well as degradative functions^49^, IL-6 demonstrates pro-fibrotic functions on cellular and systemic level^50–52^. In contrast, TNF-α induces fibrosis as a systemic trigger of inflammation, overlaying direct anti-fibrotic cellular effects^48, 53–56^. Materials’ ability to induce migration of cells to the implant is measured by IL-8 as a bi-functional chemokine that guides the infiltrations of inflammatory cells as well as fibroblasts^57–59^, rendering both, the inflammatory trigger and the cellular key mediator of fibrosis – the fibroblast. Additionally, a chemotactic potential has been furthermore reported for IL-10^60^, whereas the complete knockout demonstrated its anti-inflammatory functionality in fibrosis^61, 62^. Those controversial studies might refer to its pleiotropic concentration-dependent, stabilizing at moderate concentrations soft tissue balance, whereas its overexpression leads to fibrocyte recruitment and M2 activation, and thereby to fibrosis. A direct measurement to assess a biomaterials’ potential to induce fibrosis is the quantification of pro-fibrotic growth factors, such as TGF-ß1^63–65^.

To finally demonstrate the relevance of test conditions, a comparative model was introduced. The model facilitated the assessment of test conditions regarding their capacity to predict the fibrotic response of a biomaterial *in vivo*.

## Results

### Phenotypic characterization of pre-differentiated macrophages

**Figure 3:**
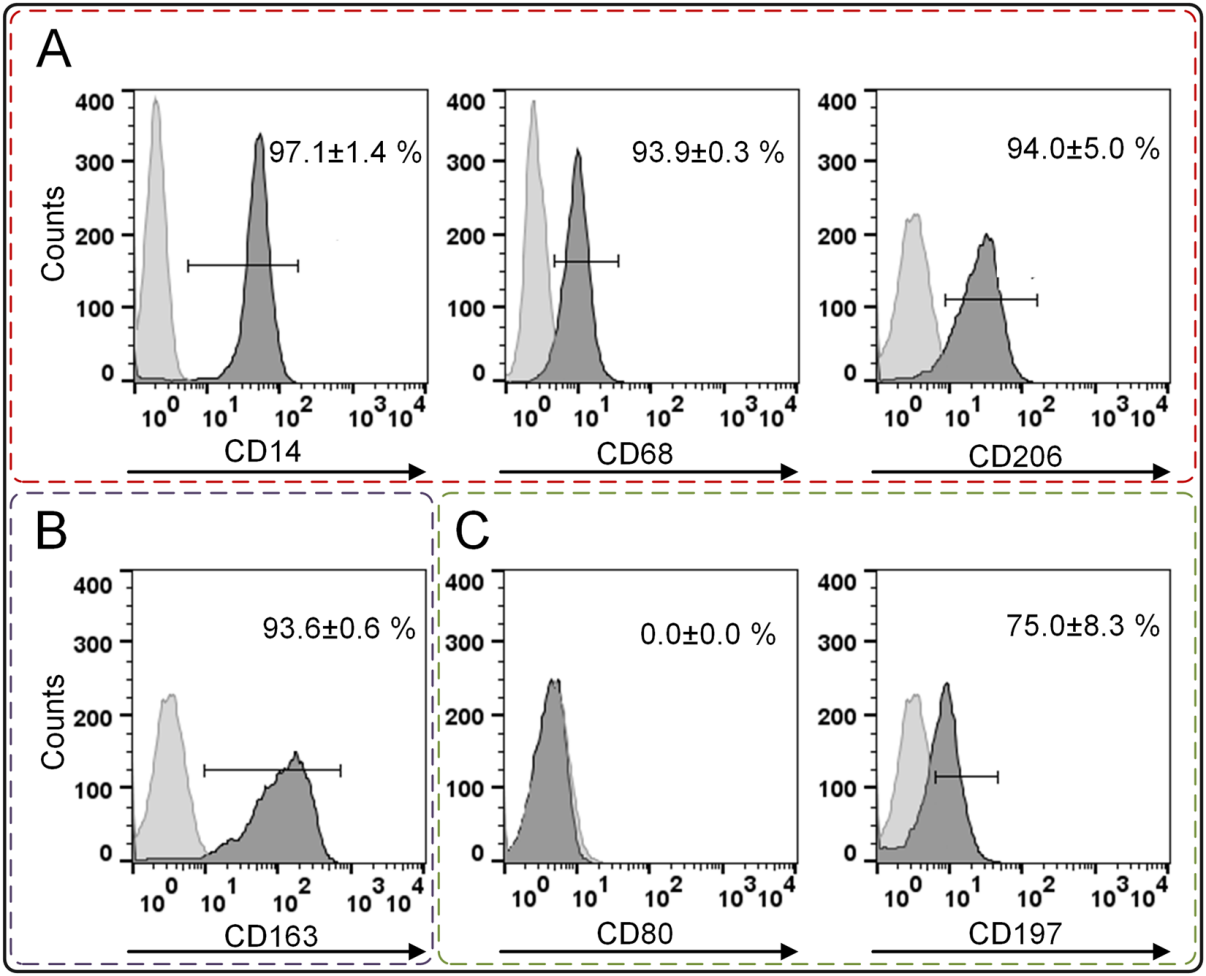
Prior material testing, the differentiation of monocytes to M2-like macrophages was confirmed. (**A**) Macrophage markers CD14, CD68, and CD206 were expressed. (**B**) The differentiation towards a M2-like phenotype was shown by CD163 positive profile, whereas (**C**) CD80 allowed distinguishing between M1 and M2. Here, its absence rejected the M1 differentiation. The marker CD197 was expressed by macrophages differentiated from whole-blood-derived monocytes. Histograms exemplarily represent the differentiation cluster of one donor; whereas mean values ± SD represent data of three blood donors. A high compliance between all three donors was found. The light-grey histograms represent the isotype controls. The following abbreviations are used: SD for standard deviation, CD for cluster of differentiation.

*In vivo*, monocytes differentiate immediately upon extravasation. Thus, our test system was based on monocyte-derived primary macrophages. Furthermore, macrophages were differentiated towards a M2-like phenotype due to its involvement in the long-term fibrotic response to biomaterials. Therefore, macrophage colony-stimulating factor (M-CSF) was applied for six days to culture medium^21, 22^. Robustness of differentiation was shown by analyzing phenotypic markers (Fig. 3). As typically expected, CD14, CD68, and CD206 were exhibited by obtained macrophages. The M2-like-phenotypic expression of CD163, in absence of M1-specific CD80 verified induced M2-like cell identity. Surprisingly, the isolation source influenced the expression of CD197. As previously described, monocytes isolated from leukocyte concentrate showed an absence of CD197 following M-CSF-induced differentiation^66^. In this study, we used whole blood to isolate and differentiate monocytes to M2-like macrophages. Thereby, we identified CD197 as a tissue-source-dependent marker. Post differentiation, macrophages were cultured on biomaterial surfaces for 48 h (experimental approach see Fig. 1).

### Macrophage morphology is influenced by test condition

**Figure 4:**
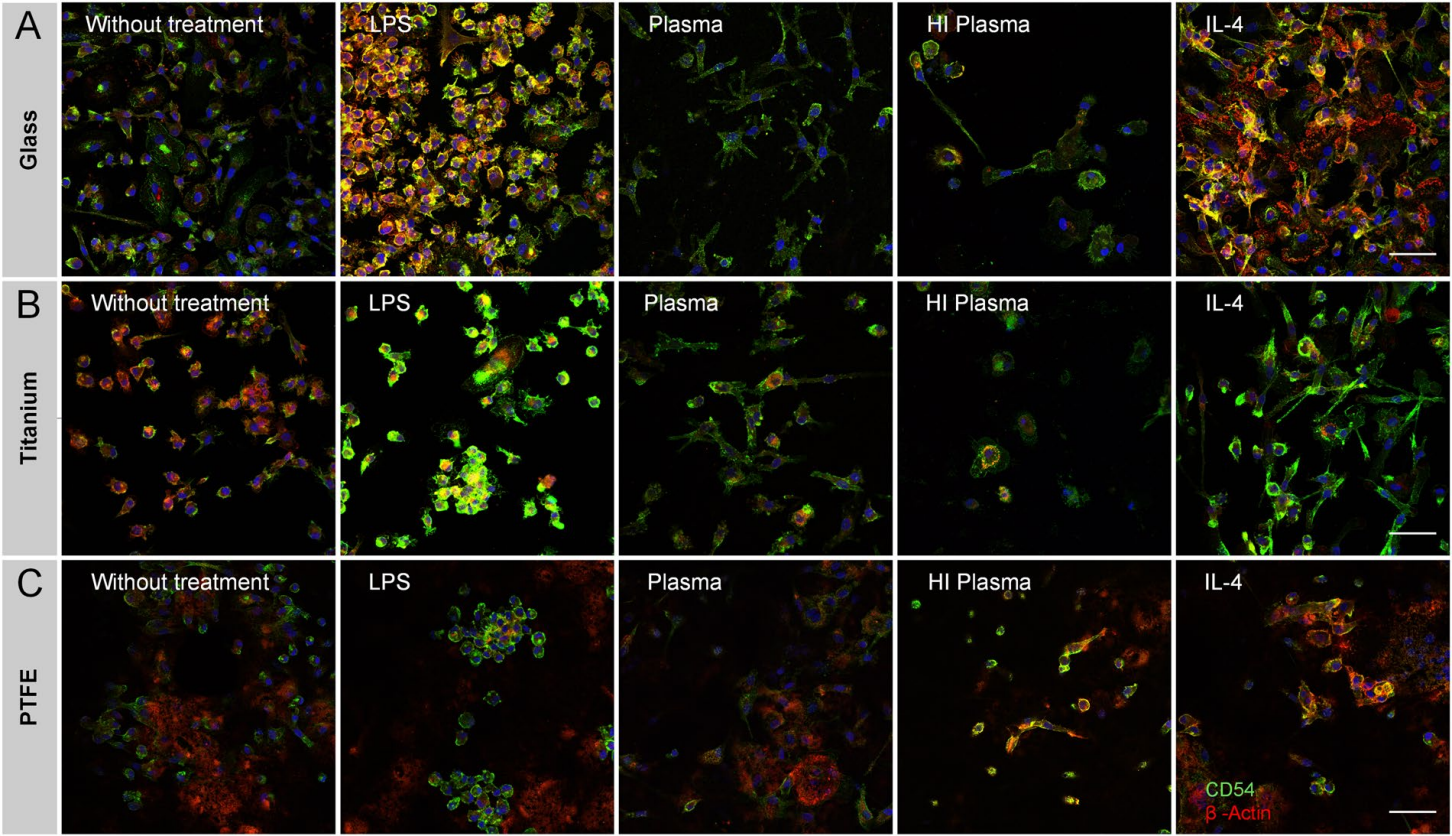
Immune-histological staining of intercellular adhesion molecule CD54 (green) and β-Actin (red) illustrated morphological changes in dependency of test conditions and material on (**A**) glass, (**B**) titanium and (**C**) PTFE. An uneven topography and a long working distance prohibited to capture sharp images on silicone and PE surfaces. The scale bar depicts 50 µm and is valid for all images. The following abbreviations are used: HI for heat-inactivated.

Glass, titanium, PTFE, silicone, and PE are commonly-used biomaterials. Moreover, these materials are known to cause a specific fibrotic response, thereby allowing the validation of the assessed test conditions regarding their predictive accuracy (see Table 1). Considering different scenarios such as inadequate cleaning of an implant, contact to blood fluid, and presence of neighboring inflammatory cells such as granulocytes and T_H_2-lymphocytes at a wound site, the assessed test conditions comprised the physiological effects of LPS contamination, contact to autologous blood plasma, and IL-4 cytokine milieu on the response of macrophages in contact to different biomaterials. To address our first hypothetical fibrotic driver - a strong cellular viability on materials’ surface - we investigated cell adherence and morphology by histologically staining CD54 and ß-Actin on glass, titanium, and PTFE (Fig. 4). Topography and working distance did not allow capturing images on silicone and PE, demonstrating a limited applicability of histological assessments that requires suitable surface geometries. Nevertheless, after 48 h of culture, a strong effect of the test conditions on cellular adherence and shape was revealed on the assessed materials. On glass, titanium, and PTFE, a circular shape of macrophages was observed without additional pretreatment. In contrast, LPS induced cellular adherence on glass and a cell clustering on titanium and PTFE. Macrophages’ formation of elongated cytoplasmic sprouts, strongly observed on glass and titanium, was affected by plasma surface treatment. As expected, surface treatment with heat-inactivated plasma led to a low cell adherence on all assessed materials. Interestingly, morphological changes of macrophages in between tested materials were highly provoked following IL-4 stimulation. On glass surfaces, a closed cellular cover was formed, whereas an elongated cellular shape was observed in response to titanium. The typically observed macrophage clustering on PTFE was not inhibited by IL-4.

### Cellular viability on biomaterials reveals an effect of test conditions

**Figure 5:**
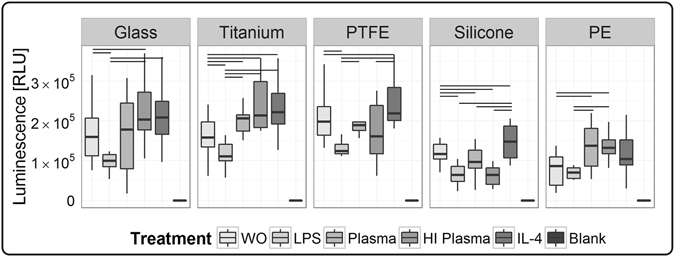
Surface-associated viability of macrophages following test procedure of 48 h was assessed by semi-quantitative ATP measurement, using a luminescence-based assay (CellTiter). Significant differences between the test conditions were found on all tested materials. Data is comprised of ten human macrophage donors (n = 10). Significance level is considered with a *p*-value ≤ 0.05. The following abbreviations are used: WO for without treatment, HI for heat-inactivated.

Based on the assumption that viable cells at wound site sustain a chronic responsiveness to the biomaterial, cell count is a well-accepted parameter to asses biocompatibility [ISO 10993-6:2007, Part 6]. As described for silicone and PE surfaces, microscopic imaging is limited to specific working distances and suitable sample geometries. Instead of histological quantification, we preferred luminescence-based ATP measurements (CellTiter) as a viability parameter (Fig. 5). This quantitative assay covers in a direct surface-exposed procedure the whole surface area, and is suitable for various surface geometries without losing cells due to extensive washing. Interestingly, cell counts did not correlate to viability assessed by luminescence. Exemplarily, luminescence readout dropped after LPS stimulation, whereas IL-4 stimulation increased viability - in both cases, a high cell adherence was observed in histology. In addition to cell counts, ATP values are influenced by macrophages’ phenotype-dependent metabolic state. Inflammatory M1 macrophages are metabolically reprogrammed to aerobic glycolysis, switching metabolism towards a faster ATP synthesis with a decreased net yield (two ATP molecules). In contrast, chronic M2 macrophages classically generate ATP by mitochondrial oxidative phosphorylation (net yield of 36 ATP molecules)^18, 67^. Our findings correspond to those expectations: on all tested materials the LPS- and IL-4-stimulated test conditions represented the minimum and maximum values of viability. Those findings support the capacity of our experimental design to cover the whole macrophage spectrum between M1 and M2 phenotypes. In addition, the impact of our study design, to evaluate test conditions is underlined by significant differences. Exemplarily, for titanium and silicone, cultures without additional treatment revealed significant differences in viability compared to all other tested conditions.

A material-dependent effect on viability was found for both plasma conditions. Following plasma treatment, the viability assessed on glass, titanium, PTFE and PE was in the higher measurement range, whereas on silicone surfaces both plasma test conditions showed values in the lower measurement range. Independent of test conditions, differences in viability between materials were observed, exemplarily demonstrated by a lower viability on silicone and PE.

### Test conditions influence cytokine secretion

**Figure 6:**
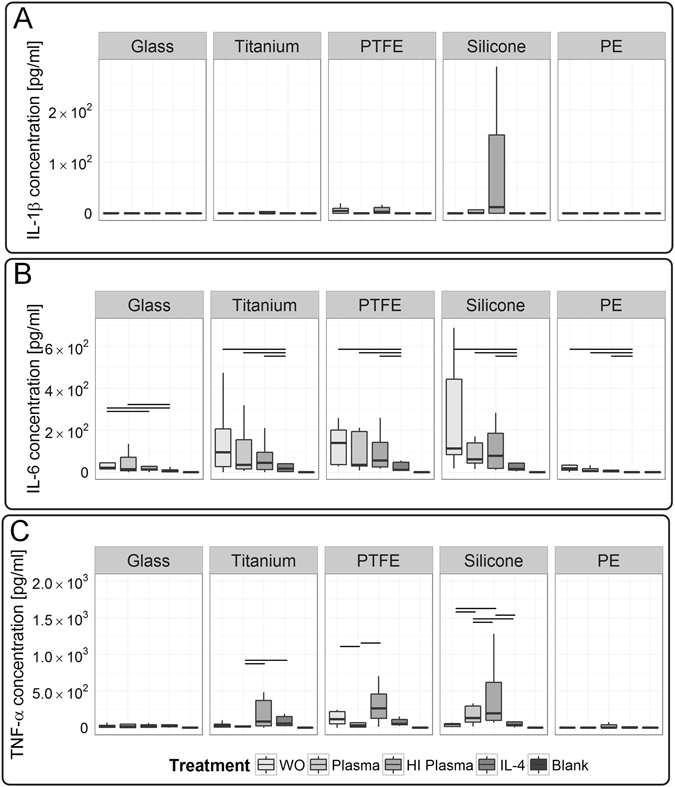
Macrophages’ response showed a dependency on test conditions and revealed a material induced secretion of acute pro-inflammatory cytokines. (**A**) Interestingly, IL-1ß did not resolve differences between test conditions. (**B**) In contrast, IL-6 showed a strong dependency on test conditions for tests on glass, whereas (**C**) TNF-α demonstrated a dependency for tests on titanium, PTFE and silicone surfaces. Data of ten donors is shown (n = 10). A p-value ≤ 0.05 is considered as significant. LPS stimulation induced a high cytokine secretion, reasonably leading to provide the obtained data in separate graphs (see Figure S1). The following abbreviations are used: WO for without treatment, HI for heat-inactivated.

The cytokine secretion by material-resident macrophages showed a high variation in between blood donors. To cover this donor-to-donor variation, we increased experimental replicates to ten blood donors. A pro-inflammatory component at the initial stage of cell-implant contact is one key mechanism mediating the macrophage-modulated chronic response to a foreign body. To address this second hypothesis, the concentrations of pro-inflammatory IL-1ß, IL-6, and TNF-α (Fig. 6) were analyzed in the supernatant at short-term 48 h. LPS stimulation increased the secretion of most cytokines to a major extent; exceptionally TGF-β1 levels remained entirely LPS-unaffected. Due to the lower cytokine levels observed for all other test conditions and to increase the resolution of test conditions, data of LPS stimulation is shown in separate graph (Figure S1).

With respect to test variables, no significant differences between materials and test conditions were detected for IL-1ß levels. On most materials macrophages’ IL-1ß secretion remained below detection limit. Similarly, also in response to PTFE and silicone only few donors secreted IL-1ß (Fig. 6A). In contrast to IL-1ß, macrophages responded condition-specifically to biomaterials by IL-6 secretion. On all tested materials, IL-4 treatment induced a decrease of IL-6. A modulatory effect between the test conditions was observed on glass, where significant higher IL-6 levels were detected under non-treated test conditions compared to heat-inactivated plasma and IL-4 treatment (Fig. 6B). Titanium, PTFE, and silicone surfaces stimulated TNF-α secretion, especially for heat-inactivated plasma. A modulatory power of plasma-material-interaction was observed on titanium and PTFE, where plasma induced a TNF-α decrease, in contrast to an increase on silicone surfaces. For TNF-α strongest dependency on test conditions was detected for the material silicone (Fig. 6C).

**Figure 7:**
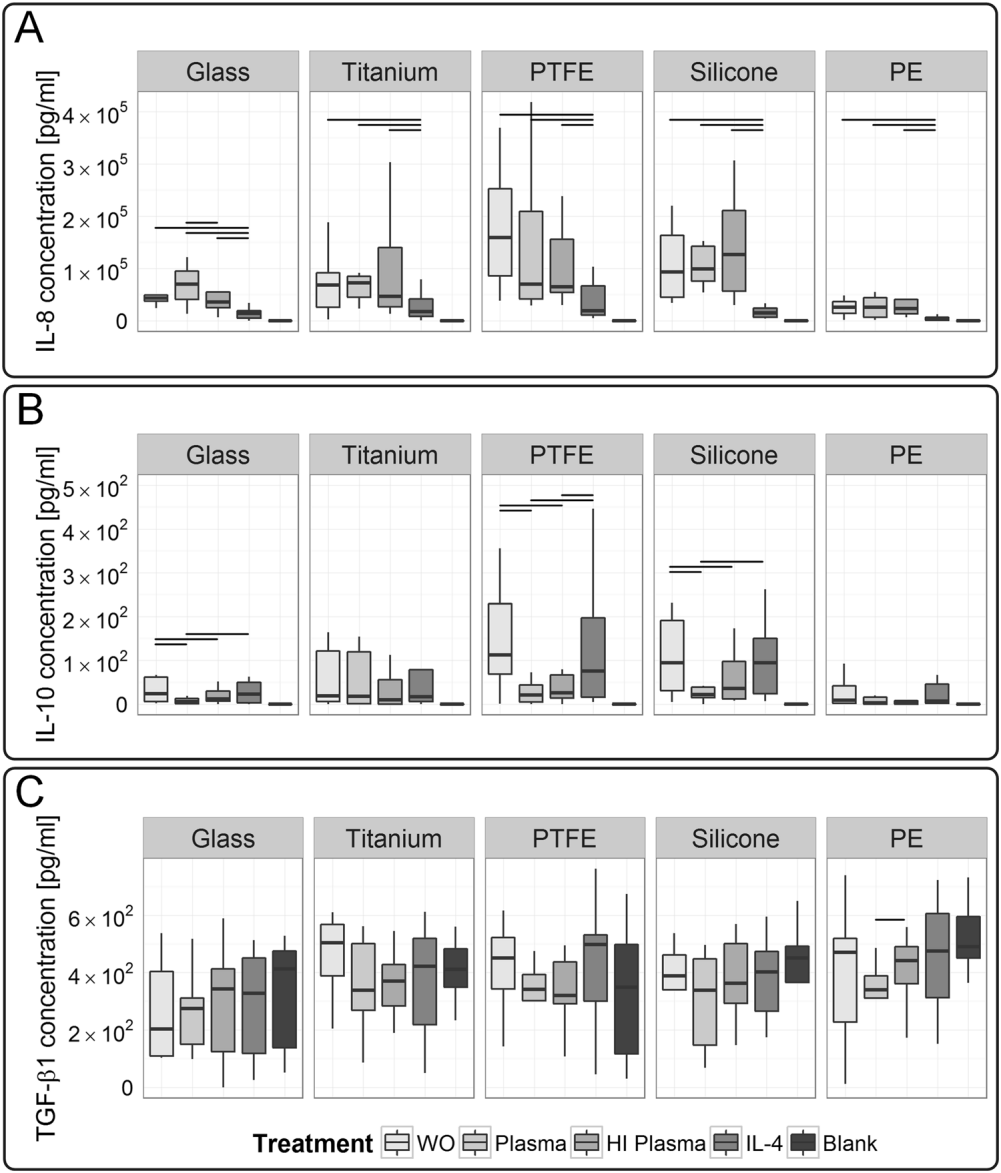
Chemokine IL-8, and anti-inflammatory cytokine IL-10 revealed significant differences between the test conditions, whereas pro-fibrotic growth factor TGF-β1 did not resolve differences. (**A**) Here, we demonstrated a test condition dependency of IL-8 levels for tests on glass. (**B**) Additionally, IL-10 levels differed significantly between test conditions on glass, PTFE and silicone surfaces. (**C**) Macrophages’ secretion of TGF-β1 did not exceed the basal level found in the culture medium, thereby no effect of the test conditions were resolved. Those cytokines were analyzed in the supernatant of ten macrophage donors (n = 10). A *p*-value ≤ 0.05 is considered as significant. LPS-induced secretion of cytokines is provided in separate graphs (see Figure S1). The following abbreviations are used: WO for without treatment, HI for heat-inactivated.

Considering our third model hypothesis - a chemokine gradient guiding further cells to implant region - we identified IL-8 as a potent chemotactic readout factor characterizing the potential of a test condition to induce the release of a chemotactic gradient by macrophages (Fig. 7A). Hereby, in response to glass a significant IL-8 increase was detected for native compared to heat-inactivated plasma test condition. Moreover, on all tested materials, IL-8 levels were significantly decreased under IL-4 stimulation.

Following differentiation, a specific cytokine profile of IL-10 (anti-inflammatory), in absence of IL-12 (pro-inflammatory), is phenotypically expected for M2-like macrophages. Moreover, IL-10 secretion depends on costimulatory activation, e.g. by LPS or material characteristics. Despite pro-inflammatory stimulation, the activated M2-like-M1-transition phenotype exhibits a locked IL-10 versus IL-12 response mode^68^. Our results are consistent with those reports, strengthening a high stability of our M2-like phenotype during the test procedure (Fig. 7B). Nevertheless, a treatment dependency of IL-10 level was found on glass, PTFE, and silicone surfaces, whereby on those materials, IL-10 levels were highest for untreated and IL-4-treated test conditions.

We included blank medium cultured on respective material surface as a reference to assess constituents of cytokines present in medium. To note, all measured cytokines in samples obtained from surfaces that were treated with native and heat-inactivated plasma did not exceed blank medium control; thereby blank medium was evaluated as a predictive control. As a component of fetal calf serum that was supplemented to the cell culture medium, TGF-β1 was present at high basal levels in the medium control (Fig. 7C; see blank medium measurement). In previous studies, we already showed that fibroblast growth factor (FGF) and platelet-derived growth factor (PDGF) did not exceed the medium threshold as well^66^. Nevertheless, in mimicry to the microenvironment at the implant site, serum supplementation to culture medium resembled the dynamics of protein adsorption to biomaterial surface^69^. Interestingly, on PE a significant TGF-β1 consumption by macrophages was detected by a decreasing TGF-ß1 concentration for native compared to heat-inactivated plasma test conditions.

Overall, significant differences in between the test conditions were demonstrated by the cytokine release. IL-6 and IL-8 showed a test condition dependency for tests performed on glass, whereas TNF-α demonstrated a test condition dependency for tests conducted on titanium, PTFE, and silicone. Additionally, differences in the IL-10 concentration were observed in between the test conditions for glass, PTFE and silicone. However, no general valid correlation in between readout factors and the test conditions was found.

### Comparative modeling allows condensation of the data to a scoring system used for biomaterial ranking

**Figure 8:**
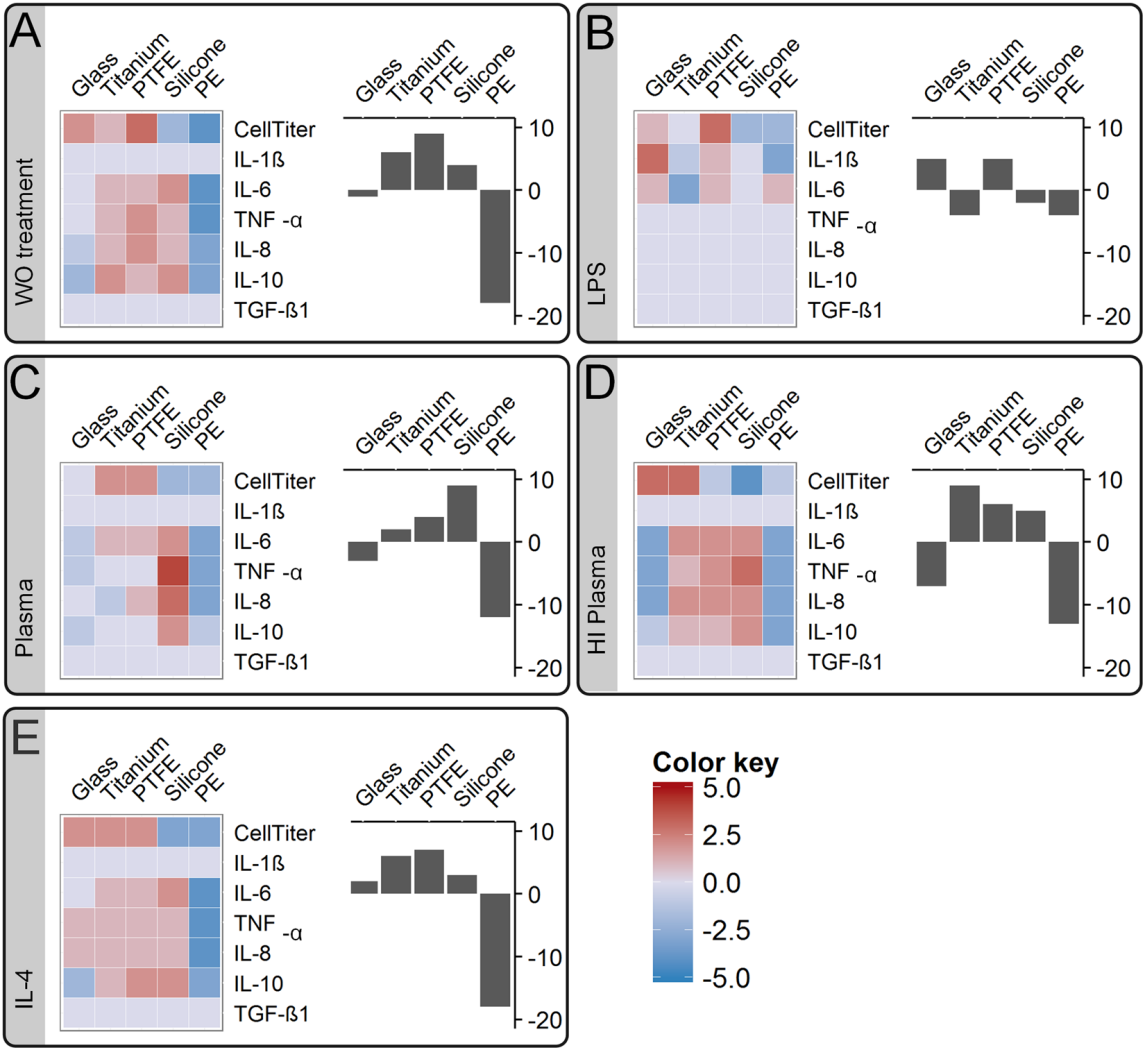
A significance profile of each material in comparison to all other tested materials was translated to a material scoring model. The obtained significance score values were translated into a color map in range of blue for minus values to red for positive values (left graph). Thereby, this scoring model visualized differences between the test conditions and finally allowed the evaluation of each specific readout factor respective to its predictive power. Finally for each material, the material ranking model summarized all readout-dependent scores in a bar plot (right graph). By comparing the literature-based biomaterial ranking to the obtained material rankings, a validation of each test condition for its accuracy to predict a fibrotic progression was performed: (**A**) without (WO) treatment, (**B**) LPS stimulation, (**C**) native plasma and (**D**) heat-inactivated (HI) plasma treatment as well as (**E**) IL-4 stimulation.

A comparative model was introduced to show the relevance of test conditions on the capacity of an *in vitro* test to discriminate in between biomaterials (Equation 1 and Figure S2). For each test condition, the sum of the significance profile from pair-wise comparison of each material to all other tested materials was derived. Thereby, materials that were significantly higher for many readout parameters compared to other tested materials were attributed with a high positive score, whereas a low scoring was assigned to materials that showed many significantly lower readout parameters. This simple scoring condensed the complex data set composed of five materials, five test conditions, ten donors, and seven readout factors to one assessment criterion. The scoring of each test material was translated into a heat map, graphically representing differences between biomaterials and for each specific readout factor (Fig. 8, heat map). Finally, by summation of all readout-factor-dependent scores a test-condition-specific material ranking was obtained (Fig. 8, bar chart).

### Plasma treatment facilitates predictive assessment of biomaterials

According to current literature (see Table 1) the potential of tested biomaterials to induce a fibrotic response *in vivo*, increases in the order of PE, titanium and PTFE to silicone. This state-of-the art material assessment was used as the basis to validate the predictive power of the selected readout factors or finally of the derived biomaterial ranking.

The predictive power of each readout factor was visualized in a heat map (Fig. 8). A readout factor with an increasing color from blue to red for PE, over titanium and PTFE to silicone, represented a promising candidate for a parameter with a high predictive power. Exemplarily, under plasma test conditions IL-8 was identified as a highly predictive factor. Whereas, IL-6, TNF-α, and IL-10 showed a high correlation too, however finally lacked a discrimination between the materials titanium and PTFE. In contrast, IL-1ß and TGF-ß1 did not resolve differences between the tested biomaterials, and thereby totally failed a correlation to the literature-based biomaterial ranking.

In our material scoring model, the color pattern of the heat map revealed differences between the significance profiles under each test condition. Those findings strengthened the importance of our experimental approach to ensure suitable test conditions when assessing a biomaterial *in vitro*. Differences of the color pattern between the test conditions finally affected materials’ ranking position.

State-of-the-art fibrotic classifications of the materials tested here allowed proving the validity of our data. Thereby, the ranking obtained under native plasma test conditions, increasing from PE (∑score = −12), titanium (∑score = 2), and PTFE (∑score = 4) to silicone (∑score = 9) was identified as a highly correlating to respective animal and clinical studies (see Table 1).

Interestingly, in analogy to plasma treatment, PE and glass were attributed with negative values for untreated and heat-inactivated plasma test conditions. Moreover, PTFE, titanium, and silicone obtained positive ranking values. However, the relative ranking position of the positively valued materials did only correspond to current biomaterial assessment for plasma treatment. This finding emphasized that especially silicone, PTFE and titanium require suitable test conditions.

LPS-dependent material scoring was limited to the readout factors CellTiter, IL-1ß, and IL-6, whereas all other readout factors revealed no significant differences between materials for LPS stimulation. Hereby, under pro-inflammatory LPS stimulation, IL-1ß entirely resolved significant differences in between materials. LPS stimulation led to a biomaterial ranking that showed no similarities to the rankings obtained under other test parameters: titanium (∑*score* = −4), silicone (∑*score* = −2) and PE (∑*score* = −4) received a negative value, glass (∑*score* = 5) and PTFE (∑*score* = 5) were attributed with positive values. Those results demonstrate how sensitive a pyrogen contamination impairs the assessment of a biomaterial.

To sum up, our material scoring model visualized differences between tested materials and between the readout factors (Fig. 8, heat map). The heat map allowed the identification of readout factors with a high predictive power. Differences between the color patterns strengthened the importance to compare test conditions and to characterize their influence when assessing a biomaterial *in vitro*. This has not been shown before. The ranking obtained under native plasma test conditions, showed a high predictive power in respect to animal and clinical studies, which is an important finding in biomaterial development. Most striking, our results demonstrate that a correlation between *in vitro* and *in vivo* biomaterial studies is attainable, if a suitable test condition is applied.

## Discussion

Despite scientific efforts to advance a materials capacity to achieve a low fibrotic encapsulation, all medical devices elicit adverse tissue responses. The current gold standard for material assessment is the animal model that is cost- and time-intensive^70^. Complementary, screening for biomaterials can be supported by *in vitro* tests. However, *in vitro* tests often entail a poor correlation between *in vitro* and *in vivo* ^19^. By comparing test conditions in a human *in vitro* biomaterial study, we identified an experimental setup that closely correlates to the fibrotic response observed in animal and clinical studies.

Post-implantation of a biomaterial, a transition of acute pro-inflammation to a chronic progression into fibrous encapsulation is observed. The complexity of this fibrotic response is a major hurdle for the development of an *in vitro* biomaterial test system. To assess a biomaterial through the secretion of cytokines that are known as molecular mediators of fibrosis, is still a relatively new field^45^. Here, we showed that on basis of multiple readout parameters the sensitivity and accuracy of an *in vitro* test system are increased. By our multi-parametric approach, test conditions that reflect a biomimetic implant scenario such as implant contamination, blood-protein-material interaction, or an immunological wound niche were successfully evaluated regarding their predictive power. Each test condition was proved by a broad spectrum of four, totally distinct, biomaterial types: titanium, PTFE, silicone, and PE. For those tested biomaterials, literature data describing materials’ fibrotic progression *in vivo* was harnessed to validate a test condition. Following this procedure, we identified the pretreatment of test surfaces with human blood plasma as a test procedure with a high predictive accuracy. In detail, by supplementation of calcium to citrate-phosphate-stabilized plasma, clotting and the formation of a fibrin network are induced. Thereby, on materials’ surface a three-dimensional fibrous niche is assembled^71^. Furthermore, through the biomaterial-dependent adsorption of plasma proteins cell adhesion is enabled, allowing the cells to sense and to respond to the foreign surface^69^. The high correlation of this biomimetic approach to the *in vivo* situation justifies novel test systems based on plasma clotting.

The screening for a test procedure with a high predictive power and accuracy is complex, like seeking a needle in a haystack. Nevertheless, a poor correlation between inflammatory animal models and human conditions strengthens its importance^72^. Critical voices have been raised, stating an overreliance of animal systems to model human immunology^73, 74^. Particularly, it has been shown, that macrophages of different tissue sites and in between species differ in their phagocytic activity, chemotactic responsiveness and sensitivity^75^. Moreover, differences between species are morphologically visible; pulmonary alveolar macrophages from mice, rats, and dogs show similarities, whereas humans’ are heterogeneous and larger in size^76^.

This data supports the importance of orientating biomaterial research on the complex conditions found in humans. The use of human cells and matrix components - here evaluated for blood-derived macrophages and fibrin matrix - is actually no translational immunological research, nevertheless represents a step towards clinical translation, which is often underestimated^74^. Therefore, the establishment of an *in vitro* test system based on human cells and tissues into current common practice needs to be promoted^74^. Due to the use of single cell lines or relatively small donor groups, *in vitro* test systems are often criticized and diminished considering their predictive power. By the use of primary macrophages from a relative large number of blood donors (n = 10), we addressed both aspects in our study. In addition, the availability of human blood allows the isolation of relatively large cell amounts, and thereby a large-scale study design becomes achievable - in this study, five different materials under five test conditions were tested. This resulted in twenty-five test approaches and a total test surface area of 225 cm^2^. The suitability of our test parameters in combination with our identified test condition, represent a basis for the development of a large-scale screening platform to identify promising biomaterials.

**Figure 9:**
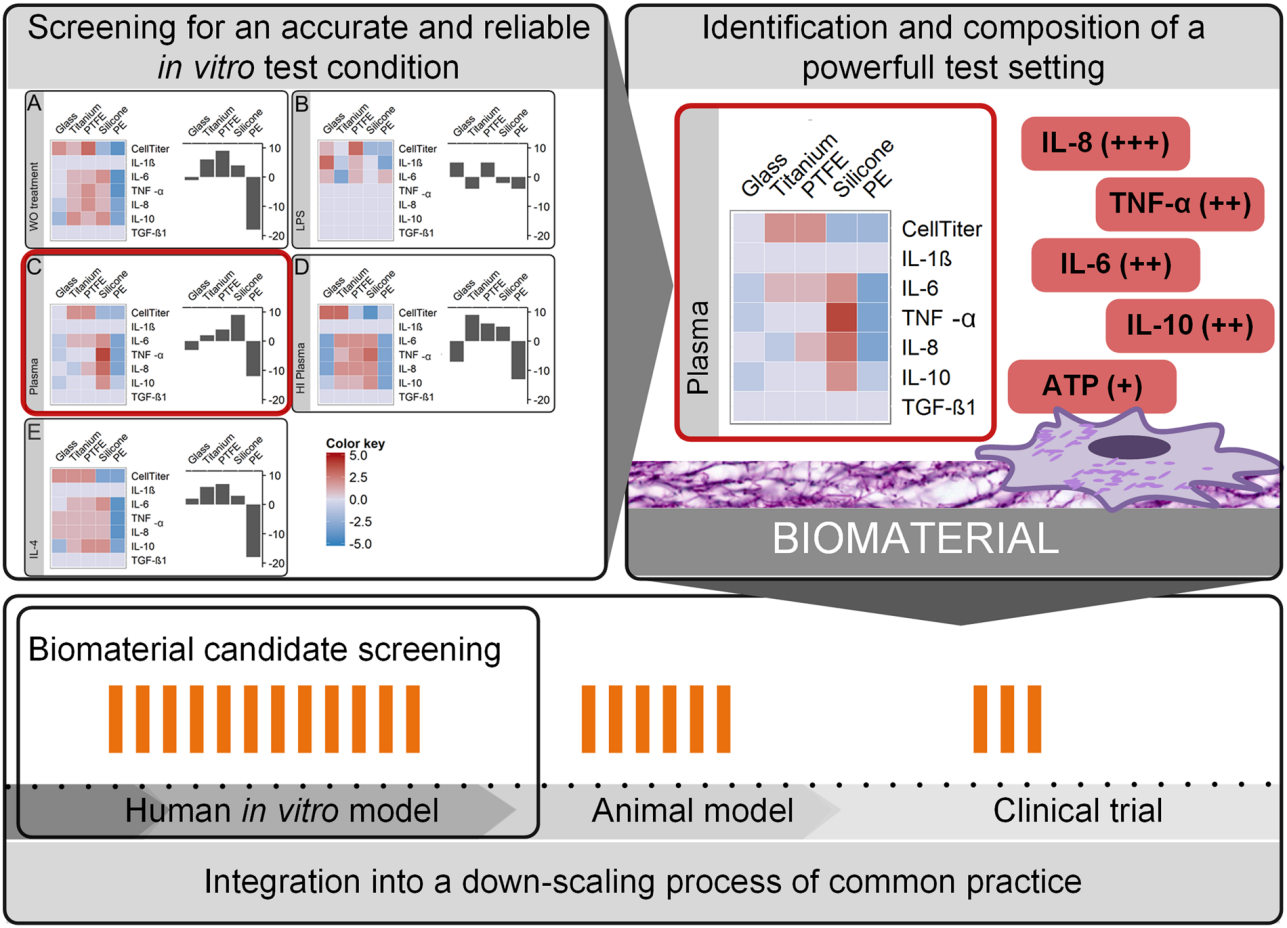
The integration of human-based *in vitro* test models into a down-scaling process of common practice is often neglected. One major flaw is a low correlation of *in vitro* tests to *in vivo* studies. In this study we propose a procedure to overcome this hurdle. In a large-scale *in vitro* screening approach, we identified an accurate and suitable condition for biomaterial testing and proved its validity by correlation to a well-described effect-the fibrotic progression - observed *in vivo*. On basis of this screening approach a well-defined *in vitro* test setting with a high predictive power was composed of (I) plasma pretreatment of biomaterials’ surface and (II) a set of highly predictive readout factors. We hope that the obtained data justifies a down-scaling of the *in vitro* test procedure to the identified condition and will be applied for large-scale biomaterial candidate screenings in following studies. Finally, followed by current common practice to study promising candidates in systemic animal models, and carrying the positively-proofed biomaterials in human subjects.

Independently on the implant site, all implantation procedures have one in common; due to the invasiveness a bleeding process is induced, and the implant surface gets in direct contact to blood. This strengthens our approach to study blood-component-material interactions. However, blood-component-interactions do not reflect a tissue-side-specific response or systemic effects. The tissue response at different implantation sites may also be divergent^77^, especially in highly vascular areas such as bone and muscle, or avascular areas such as cartilage. Thereby, the clinical relevance of the *in-vitro*-generated data of promising biomaterial candidates is not necessarily guaranteed. Thus, the data needs to be proved in animal models to make a decision which biomaterial candidate is carried forward into clinical trials. Our study emphasizes the potential of human-based *in vitro* tests to correlate with *in viv*o studies under suitable test conditions – here demonstrated for native plasma surface pretreatment. Thus, we propose the integration of human-based *in vitro* test systems into common practice for biomaterial assessment (Fig. 9): Starting with a broad *in vitro* screening for a predictive test condition and proving its validity by correlating to a well-described effect observed *in vivo*. Secondly, reducing the scope of test procedure to the identified condition and applying it for a biomaterial candidate screening. This is followed by the transfer of promising candidates into systemic animal studies, and finally studying the positively-proved biomaterials in human subjects.

To further improve the applicability of our test procedure, a suitable cell line can be used instead of primary human macrophages, thereby reducing the number of replicates. However, the inflammatory spectrum of cell lines is often not well-described, and must thus be compared to the response of human primary macrophages to a biomaterial. Our data set for ten different macrophage donors represents a reference to identify an appropriate cell line. Furthermore, the comparative model allowed the identification of high predictive readout factors. Thus, we will further optimize the set of readout factors by screening for promising candidates and by replacing less predictive cytokines from the experimental set up.

In general, it should be considered that all *in vitro* test systems represent models providing a specific range of physiological functions. Thus, a suitable test system to address a specific scientific question must be carefully selected. Then, *in vitro* test systems can typically serve as a screening platform that allows the identification of adequate properties prior to the assessment of most promising candidates *in vivo*. By complementation of existing *in vivo* models, such an approach follows the Russell and Burch’s 3R aspect of reducing animal burden^20, 78^. However, a poor correlation between *in vitro* and *in vivo* assessments confirms a clear need for predictive *in vitro* biomaterial tests^19^. The inadequacy of the current *in vitro* assessment strengthens our comparative approach to identify predictive test conditions for the development of a novel biomaterial *in vitro* test platform. In our human-based screening study, we demonstrated how sensitive *in vitro* biomaterial assessments rely on test conditions, finally influencing the outcome of a biomaterial ranking. Most important, we showed a high correlation in between an *in vitro* testing based on autologous plasma to *in-vivo*-obtained biomaterial assessments. Thereby, we strengthen further developments for a biomimetic test system based on the combination of a three-dimensional fibrin matrix and primary macrophages to identify promising biomaterials.

## Methods

### Ethical clearance statement

Peripheral blood samples of 10 donors were obtained under informed consent according to ethical approval granted by the institutional ethics committee of the Julius-Maximilians-University Wuerzburg (vote 182/10) from Bavarian Red Cross blood donation service (Blutspendedienst des Bayerischen Roten Kreuzes, München, Germany). Experiments with blood samples were conducted in compliance with the rules for investigation on human subjects, as defined in the Declaration of Helsinki.

### Plasma isolation from whole blood

Citrate-buffered autologous plasma was separated from human whole blood by centrifugation at 2500 x standard gravity (g_0_) for 15 min. For each donor, partial volumes of the obtained human plasma were heat inactivated for 1 h at 56 °C (Thermocycler, Eppendorf, Hamburg, Germany). To remove precipitates, a centrifugation for 1 min at 20817 × g_0_ was performed. Heat-inactivated and native plasma was stored at −80 °C. To purify autologous peripheral blood mononuclear cells, buffy coat layer was collected on top of the erythrocytes and immediately further processed.

### Isolation and differentiation of human monocytes to macrophages

Mononuclear cells from peripheral whole blood were isolated by ficoll gradient centrifugation (GE Healthcare, Freiburg, Germany). To purify monocytes from the generated cell fraction, a negative magnetic cell separation was performed (Miltenyi Biotec, Bergisch Gladbach, Germany), catching T cells, NK cells, B cells, dendritic cells, and basophils. Obtained monocytes were cultured in RPMI GlutaMax (Gibco, Carlsbad, USA) plus 10 % fetal calf serum (41F1142K, Gibco) at a concentration of 1*10^6^ cells per ml and a density of 1.5*10^5^ cells per cm^2^ on standard polystyrene cell culture dishes (TPP, Trasadingen, Switzerland). Monocytes were differentiated using 40 ng per ml recombinant human M-CSF (Peprotech, New Jersey, United States) for 6 days^68, 79^. To boost differentiation, medium was refreshed on third day of culture. On day 6, cells were harvested by mechanical cell scraping.

### Flow cytometric analysis

On day 6 following differentiation, differentiation robustness was ensured by flow cytometry. Expression profile was evaluated by antibody staining of 2*10^5^ macrophages per antigen: CD14 (555-397, BD Bioscience, Heidelberg, Germany), CD68 (11-0689-42, eBioscience, Frankfurt am Main, Germany), CD163 (12-1639-42, eBioscience) and CD206 (12-2069-42, eBioscience), CD80 (12-1639-42, eBioscience) and CD197 (130-093-621, Miltenyi Biotec). Cells were analyzed in FACS Calibur flow cytometer (BD Biosciences) and data was further processed in FlowJo software (Tree Star, Ashland, United States). Isotype controls are included in the data analysis as light-grey histograms. Cell debris were excluded from the data analysis by gating.

### Preparation of test materials

Titanium, glass, and PTFE samples were prepared as described previously^66^. Briefly, titanium films were in-house deposited on glass bowls (34 mm diameter, Brandt, Wertheim, Germany) by radio frequency magnetron sputtering using a titanium target (120 mm diameter, 10 mm height) with a target-to-substrate distance of 100 mm. PTFE layer (Rhenolase MK I-grau) was deposited on glass bowels by Rhenotherm GmbH (Kempen, Germany). Silicone test chambers were prepared in-house by using Biopor® AB clearX 70 shore (Dreve Otoplastik GmbH, Unna, Germany). To sterilize test samples prior testing, glass and silicone as well as titanium- and PTFE-coated bowls were incubated in an ultrasonic bath with deionized water for 30 min, subsequently incubated in 70 % ethanol for 15 min and finally autoclaved. Sterile standard cell culture PE culture dishes (Ibidi GmbH, Martinsried, Germany, 81156) were included in the test setting. To histologically stain cells on surfaces, glass 8-well Nunc^®^ Lab-Tek™ Chamber Slide™ (VWR International GmbH, Darmstadt, Germany) were used or PTFE- and titanium-coated glass slides (Icefrost 76 × 26 × 1 mm, Menzel, Germany) were combined with the chamber system removed from an 8-well Nunc^®^ Lab-Tek™ Chamber Slide.

### Plasma treatment of material surface

Human native and heat-inactivated plasma was thawed for 15 min at 37 °C. Plasma was applied on biomaterials’ surface in a volume per surface ratio of 50 µl per cm^2^. To eliminate the effect of the citrate buffer in the whole blood sample and thus to allow coagulation on sample surface, plasma was calcified by supplementing 150 mM calcium chloride (Sigma Aldrich, München, Germany) to a final concentration of 15 mM. Human plasma was homogeneously distributed on biomaterials’ surface and incubated for 30 min at 37 °C.

### Macrophage seeding on material surface

For evaluation of physiological variables on macrophages’ inflammatory response to biomaterials, macrophages were seeded on (I) untreated, (II) plasma treated and (III) biomaterials treated with heat-inactivated plasma. For macrophage polarization, macrophages were stimulated towards an (IV) inflammatory response by adding 100 ng per ml LPS (L4391, Sigma Aldrich) or (V) a M2-phenotype was strengthened by 20 ng per ml IL-4 (200-04, Peprotech). To evaluate the impact of medium-material interaction, (VI) blank RPMI GlutaMax media supplemented with 10 % fetal calf serum incubated on each material surfaces served as control. Macrophages were seeded on biomaterials at a cell density of 3*10^4^ cells per cm^2^ at a total medium volume surface ratio of 0.22 ml per cm^2^ in RPMI GlutaMax supplemented with 10 % fetal calf serum. All samples were incubated for 48 h at 37 °C and 5 % CO_2_ conditions. The medium was harvested at 48 h and centrifuged for 5 min at 10.000 × g_0_.

### Immunohistochemical staining

Immunohistochemical staining was performed on biomaterial surfaces following a standard staining protocol. In short, cells were fixed with 4 % paraformaldehyde (Carl Roth, Karlsruhe, Germany) for 10 min at room temperature. Primary anti-human CD54 (AH55411, Invitrogen, Carlsbad, United States) was incubated in a 1:100 dilution overnight at 4 °C and secondary anti-mouse IgG Alexa Fluor 488 (A-21202, Invitrogen) was applied in a 1:400 dilution for 60 min at room temperature. Following, 2.5 % phalloidin anti-beta-actin Alexa Fluor 555 (A34055, Invitrogen) in phosphate buffered saline plus 1 % bovine serum albumin (Applichem, Darmstadt, Germany) was incubated for 20 min. Dapi-Fluoromount (eBioscience) was used for sealing. Images were captured on a confocal laser scanning microscope (TCS SP8, Leica Microsysteme Vertrieb GmbH, Wetzlar, Germany). Background subtraction and contrast enhancement was equally performed on all images (ImageJ 1.49 m, National Institutes of Health, Bethesda, United States). Surface topography and long distance prohibited to capture images of cells on silicone and PE surfaces.

### Cell viability

Biomaterials’ surface was washed once with phosphate buffered saline (Gibco). CellTiter reagent (Promega, Manheim, Germany) was applied to a total volume of 78 µl per cm^2^ and assay was performed according to manufactures procedure. Luminescence was measured in triplicates using a microplate Tecan Reader infinite® M200 (Crailsheim, Germany).

### Cytokine measurement

Secretion of human TGF-β1 (BMS249, eBioscience) was analyzed by ELISA, used according to the manufacturer’s protocol. Samples were measured in duplicates with a Microplate Reader (Tecan Reader infinite® M200, Tecan, Crailsheim, Germany). IL-1ß, IL-6, IL-8, IL-10, IL-12 and TNF-α in cell culture supernatant were characterized by human Inflammatory Cytokine CBA (551811, BD Biosciences, Heidelberg, Germany) according to manufacturer’s protocol. Analysis was performed with FACS Calibur (BD Biosciences) and data was processed using FCAP Array Software 3.0 (BD Biosciences).

### Statistical Analysis on treatment dependency

Continuous donor-dependent data was identified as not-normally distributed using Shapiro-Wilk Test. Friedman’s ANOVA was applied, followed by Wilcoxon signed rank test to test pair-wise on significant differences between test conditions of biomaterial surfaces. For all statistical tests, a *p*-value ≤ 0.05 was considered as significant. All statistical tests were performed with OriginPro 9.1G statistical software (OriginLab Corporation, Northampton, US).

### Statistical Analysis on material dependency and biomaterial scoring

To allow a pair-wise material evaluation, a scoring system was introduced. Therefore, significant differences between two sets of *n* measured readout factors *f* for two materials *M1* ${\{{f}_{M1,i}\}}_{i=1}^{n}$ and *M2* ${\{{f}_{M2,i}\}}_{i=1}^{n}$were analyzed by Wilcoxon signed rank test. For all statistical tests a *p*-value ≤ 0.05 was considered as significant. A pair-wise significant higher mean value for material *M1* in comparison to material *M2* was translated into a scoring with a value of 1 for material *M1*, whereas a pair-wise significant lower mean value for material *M1* in comparison to material *M2* resulted in a scoring of −1 for material *M1*. If no significant difference was found (*p*-value > 0.05), a zero value was included in the assessment.

1 $$Score:=({\{{f}_{M1,i}\}}_{i=1}^{n},{\{{f}_{M2,i}\}}_{i=1}^{n})\to \{\begin{array}{cc}0, & p > 0.05\\ 1,\bar{{\{{f}_{M1,i}\}}_{i=1}^{n}} > \bar{{\{{f}_{M2,i}\}}_{i=1}^{n}}and & p\le 0.05\\ -1,\bar{{\{{f}_{M1,i}\}}_{i=1}^{n}} < \bar{{\{{f}_{M2,i}\}}_{i=1}^{n}}and & p\le 0.05\end{array}$$

Hereby, $\overline{{\{{f}_{M1,i}\}}_{i=1}^{n}}$ denotes the mean value of set of *n* measured readout factor *f* for *M1* and $\overline{{\{{f}_{M2,i}\}}_{i=1}^{n}}$ for *M2* respectively. To derive the relevance of a specific readout factor, the sum of the significance profile from pair-wise scoring for each material was calculated. Thereby, the factor-dependent relation of a material in comparison to all other materials was obtained. Final material ranking was based on all measured readout factors *f*. Therefore, the sum of all factor-dependent scores was computed.

## Electronic supplementary material

Supplementary Figure S1 and S2
