# Supplementary material for: A comparative multi-parametric in vitro model identifies the power of test conditions to predict the fibrotic tendency of a biomaterial
Source: Sci Rep. 2017 May 10;7:1689. doi: 10.1038/s41598-017-01584-9 (PMC5431855; doi:10.1038/s41598-017-01584-9)
Supplement: Supplementary file 1 — Supplementary Figure S1 and S2 [file 41598_2017_1584_MOESM1_ESM.pdf]

„A comparative multi-parametric *in vitro* model identifies the power of test conditions to predict the fibrotic tendency of a biomaterial“

Maren Jannasch[1], Sabine Gaetzner[1], Tobias Weigel[1], Heike Walles[1,2], Tobias Schmitz[1], Jan Hansmann[1,2*]

[1] University Hospital Wuerzburg, Department Tissue Engineering and Regenerative Medicine (TERM), Roentgenring 11, 97070 Wuerzburg, Germany

[2] Translational Center Wuerzburg ´Regenerative Therapies in Oncology and Musculoskeletal Disease`, 97070 Wuerzburg, Germany

*Corresponding author:

Dr.-Ing. Jan Hansmann, University Hospital Wuerzburg, Roentgenring 11, 97070 Wuerzburg, Germany, telephone number +49 931 3181209,

Jan.Hansmann@uni-wuerzburg.de

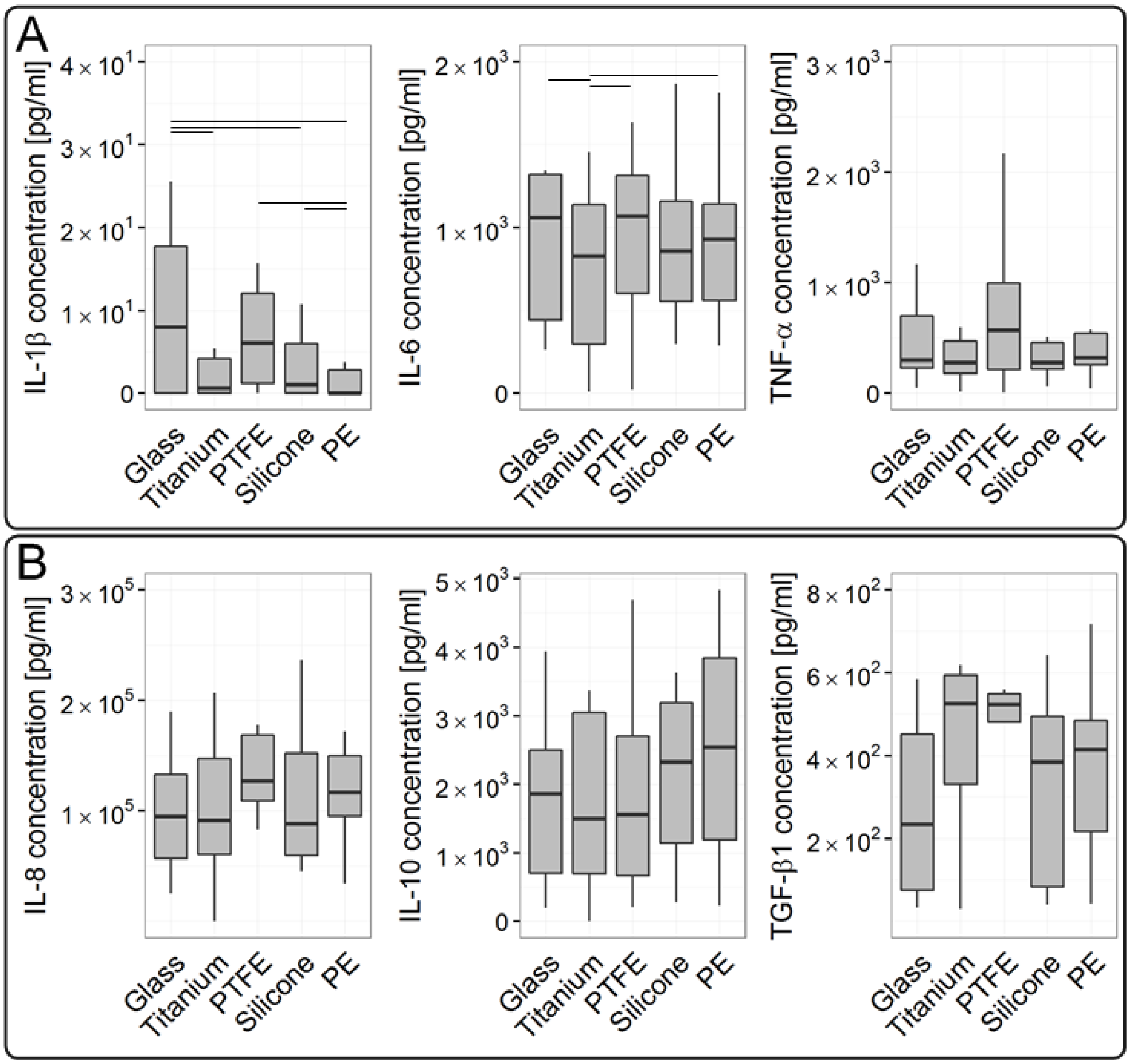

Figure S1: Pro-inflammatory LPS stimulation of macrophages induced a highly increased secretion of most cytokines compared to material-induced secretion; exceptionally TGF-β1 levels entirely remained LPS-unaffected. (A) Significant differences between materials were found for IL-1ß and IL-6, whereas no differences were shown for TNF-α and (B) IL-8, IL-10 and TGF-ß1. Supernatants of ten macrophage donors (n = 10) were analyzed. A *p*-value ≤ 0.05 is considered as significant.

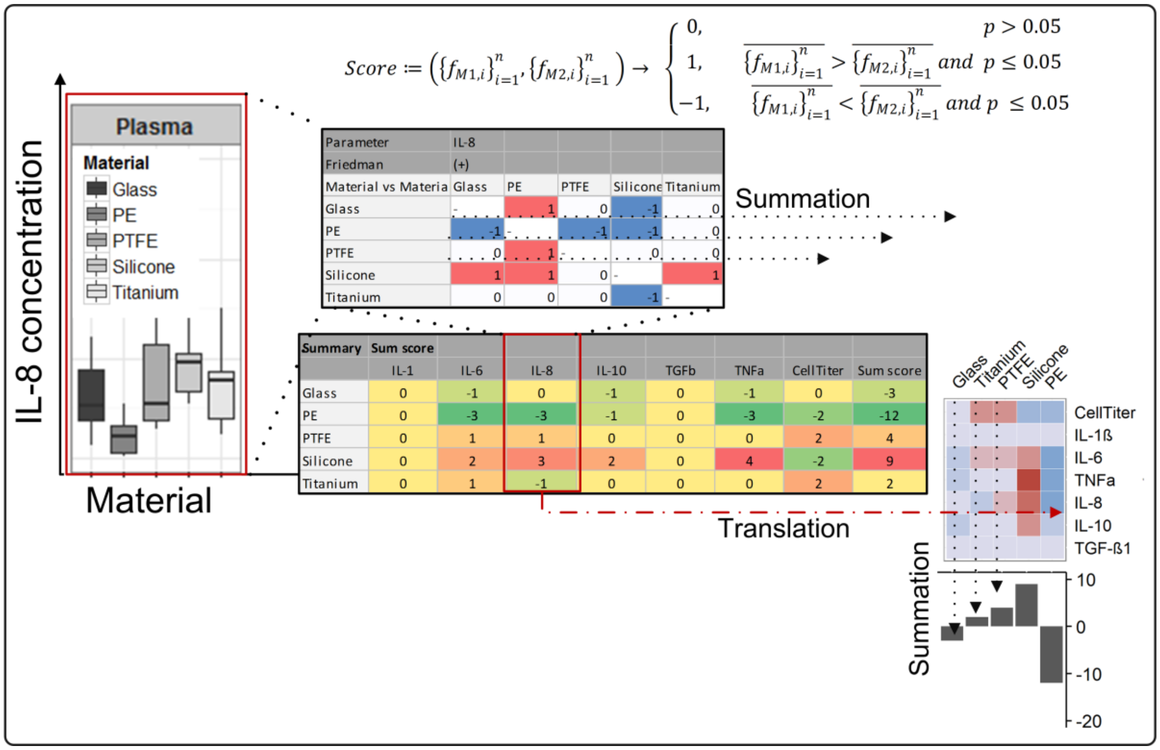

Figure S2: A scoring system was introduced allowing materials' evaluation. Therefore, under each test condition significant differences between two pairs of tested materials were translated into a scoring with a value of 1 for the significantly higher pair and a scoring of -1 for significantly lower pair (*p*-value ≤ 0.05). If no significant difference was found (*p*-value > 0.05), a zero value was included in the assessment. For each material, the relevance of a specific readout factor was derived by summation of the significance profile from pair-wise scoring. The obtained factor-dependent relation of a material in comparison to all other materials was visualized in a heat map. To generate a material ranking based on all measured readout factors, finally the sum of all factor-dependent scores was computed.
